# A dimensional warehouse for integrating operational data from clinical trials

**DOI:** 10.1093/database/baz039

**Published:** 2019-04-03

**Authors:** Michael A Farnum, Lalit Mohanty, Mathangi Ashok, Paul Konstant, Joseph Ciervo, Victor S Lobanov, Dimitris K Agrafiotis

**Affiliations:** Covance, the Drug Development Division of LabCorp, Carnegie Center, Princeton, NJ, USA

## Abstract

Timely, consistent and integrated access to clinical trial data remains one of the pharmaceutical industry’s most pressing needs. As part of a comprehensive clinical data repository, we have developed a data warehouse that can integrate operational data from any source, conform it to a canonical data model and make it accessible to study teams in a timely, secure and contextualized manner to support operational oversight, proactive risk management and other analytic and reporting needs. Our solution consists of a dimensional relational data warehouse, a set of extraction, transformation and loading processes to coordinate data ingestion and mapping, a generalizable metrics engine to enable the computation of operational metrics and key performance, quality and risk indicators and a set of graphical user interfaces to facilitate configuration, management and administration. When combined with the appropriate data visualization tools, the warehouse enables convenient access to raw operational data and derived metrics to help track study conduct and performance, identify and mitigate risks, monitor and improve operational processes, manage resource allocation, strengthen investigator and sponsor relationships and other purposes.

## Introduction

One of the main factors driving the inefficiencies in the current clinical development model is the lack of convenient and integrated access to clinical trial data. The pharmaceutical industry has struggled with data integration for many years and, despite having made significant investments, has yet to come up with effective solutions. While some worthy endeavors have been reported throughout the years (both commercial and home grown), the inherent breadth and diversity of clinical trial data, the variety or lack of standards in capturing that data in the source systems and the insular culture of the industry have been an impediment to disseminating and leveraging best practices.

Clinical trial data are typically collected through multiple systems developed by different vendors using different technologies and data standards. Conceptually, these data fall into two main categories: (i) operational data, which are used to monitor the progress and operational health of the study and (ii) patient data, which are used to assess the safety and efficacy of the therapy under investigation. For operational ends, one approach that has been used to integrate these disparate systems is through the clinical trial management system (CTMS). The CTMS is a core transactional system that is used to manage sites, monitoring visits, site issues, trip reports and various other study metrics and milestones. While certainly useful, these efforts have focused mostly on minimizing duplicative data entry rather than enabling downstream analytics (1, 2).

Data-warehousing efforts for healthcare applications have been inspired by the benefits seen in other domains, including standardization across data feeds, enhanced support for analytics and reporting and reduced burden on transactional systems (3). A substantial fraction of the relevant literature is concerned with hospital or real-world evidence data used for follow-up analysis (4–8). For clinical trial data, the number of published reports is surprisingly limited. Despite being of great interest to pharmaceutical companies and contract research organizations (CROs), methods and outcomes have typically been kept proprietary and internal to the authoring company, with some exceptions (9). This is also reflected in the lack of published standards for conforming and sharing data. While years of effort have gone into standards in working groups such as CDISC and HL7, efforts for comprehensive operational information for clinical trials are still in the early stages (10). It is indicative that the newly formed Align Clinical CRO consortium is currently in the process of drafting specifications for reporting operational metrics (11). While this will be very helpful for standardizing common exports, the initial version is not expected to capture the complete variety of operational data.

**Figure 1:**
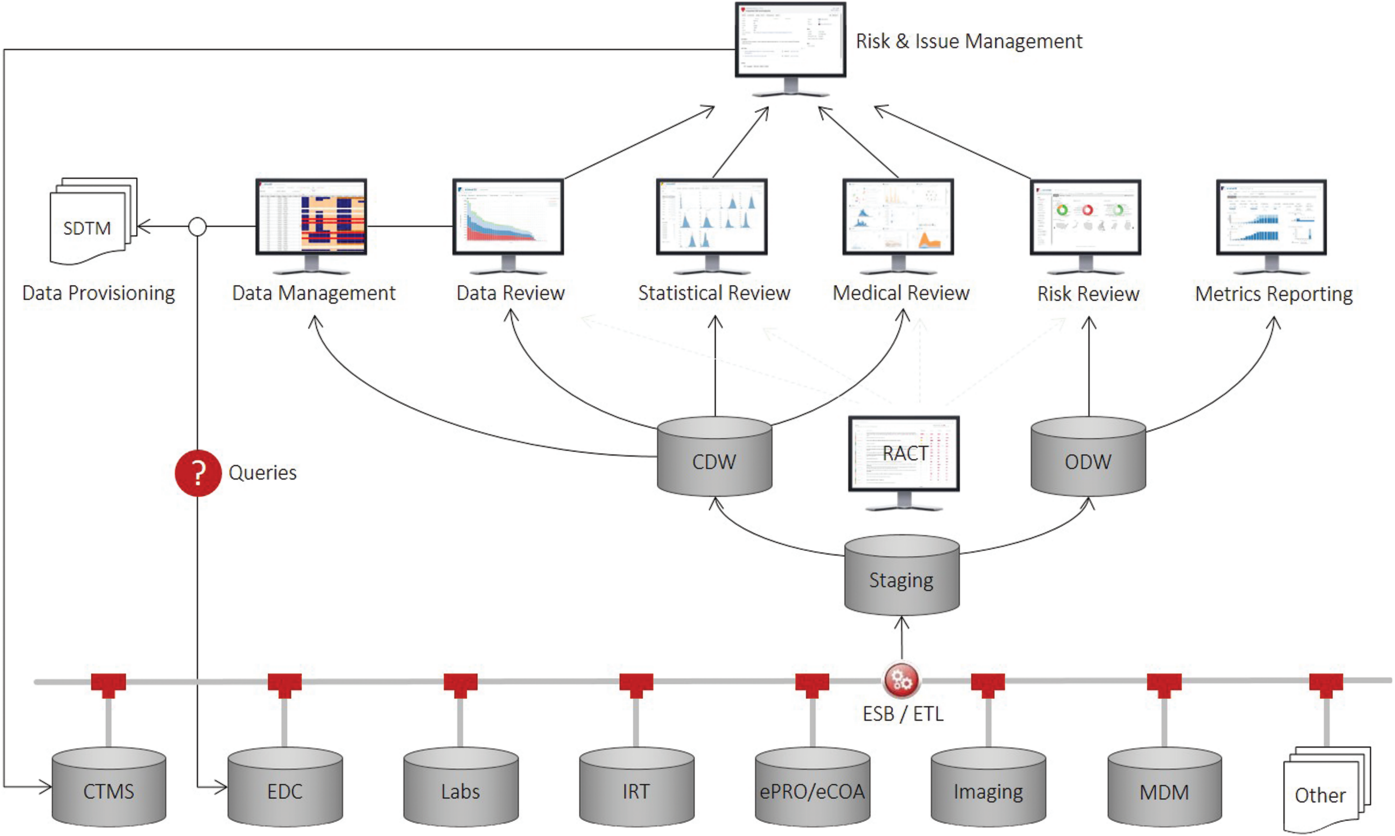
Xcellerate architecture.

Recognizing this unmet need and inspired from previous work in discovery (12), clinical (13, 14) and outcomes research (15), we have developed a comprehensive application suite, known as Xcellerate, that uses advanced data integration, analytics and visualization capabilities to improve patient safety, data quality and protocol compliance throughout the clinical development process and enable greater transparency and oversight of study conduct and performance (16, 17). As illustrated in Figure 1, the solution consists of a number of end-user applications connected to a clinical data repository that supports near-real-time acquisition, mapping and integration of clinical trial data from any germane source, including CTMS, electronic data capture (EDC) systems, interactive response technologies (IRT), electronic patient reported outcomes and clinical outcome assessments systems and electronic data transfers provided by third parties such as central laboratory, biomarker and imaging vendors. At the core of this repository are two data warehouses that store the operational and subject-level data collected during a clinical trial: (i) the operational data warehouse (ODW) that stores all the operational data and other ancillary databases and tools to support the calculation of metrics, key performance, quality and risk indicators (KPIs, /KQIs, KRIs, respectively) and triggers, thresholds and aggregation functions used to determine risk levels and monitoring interventions for risk-based monitoring (RBM); and (ii) the clinical data warehouse (CDW) that stores all the patient data in a unique NoSQL (No Structured Query Language) (18) architecture and other ancillary databases and tools to support medical, statistical and data review, safety signal detection and other analytic and reporting needs.

Our approach is based on the principle of separation of concerns, that is, the uncoupling of operational and clinical objectives. Operational objectives are focused on achieving optimal execution of clinical studies from a data quality, patient safety, timeline and cost perspective. By contrast, clinical objectives are focused on enrolling qualified patients, ensuring that the collected data are ‘fit for purpose’, and monitoring drug-related safety issues. Here, we describe the inner workings of the ODW and its supporting tools. The CDW is described elsewhere (19).

## Methods

A key feature of our system is the automated assembly and normalization of all information about a clinical trial. We have decided to divide our solution into two separate repositories (ODW and CDW) because of the vastly different nature of the data that is being stored in each system. The ODW gathers operational metrics, KPIs, KQIs and KRIs from a variety of sources, including CTMS, EDC and other systems managing laboratory tests, protocol deviations, audit findings and other types of data. These data are retrieved daily from their original sources through direct connection to the databases behind the systems (e.g. CTMS), standard web service calls such as those provided by the leading EDC vendors, file-based approaches or other methods, as described below.

### Data model

For late phase trials, the bulk of the information required for operational reporting and site monitoring comes from a relatively small number of systems that are fairly standard in the types of data they capture. Virtually, every modern clinical trial uses a CTMS to manage sites, site-monitoring visits, site issues and trip reports; an IRT to track subject enrollment, subject visits and drug allocation; an EDC to facilitate patient data collection, source data verification and data query management; an electronic trial master file (eTMF) to track documentation compliance; and several others. For some early phase trials, some of these systems may be missing altogether (e.g. IRT) or replaced with a different type of technology (e.g. bedside data capture instead of EDC). Despite the variety of different solutions on the market, most of them are designed to capture very similar data and differentiate themselves in system capabilities, user interface (UI) and ease of configuration and management.

In order to enable consistent and efficient study oversight, we have developed a unifying operational data model that represents operational entities, their attributes and their relationships. This model can support both routine reporting and monitoring needs, as well as *ad hoc* reporting and trend analytics. It is designed as a snowflake schema with shared dimensions representing key object attributes such as study, site, subject, contact, address, etc. and two types of fact tables: aggregated metrics for routine reporting and individual events (e.g. payment activities) for *ad hoc* reporting and trend analysis. Wherever appropriate, we aggregate information at various levels such as subject, site, country, study and account (study sponsor), and we provide source-specific versions of metrics to improve data quality and support operational activities (e.g. IRT enrollment versus EDC enrollment).

**Figure 2:**
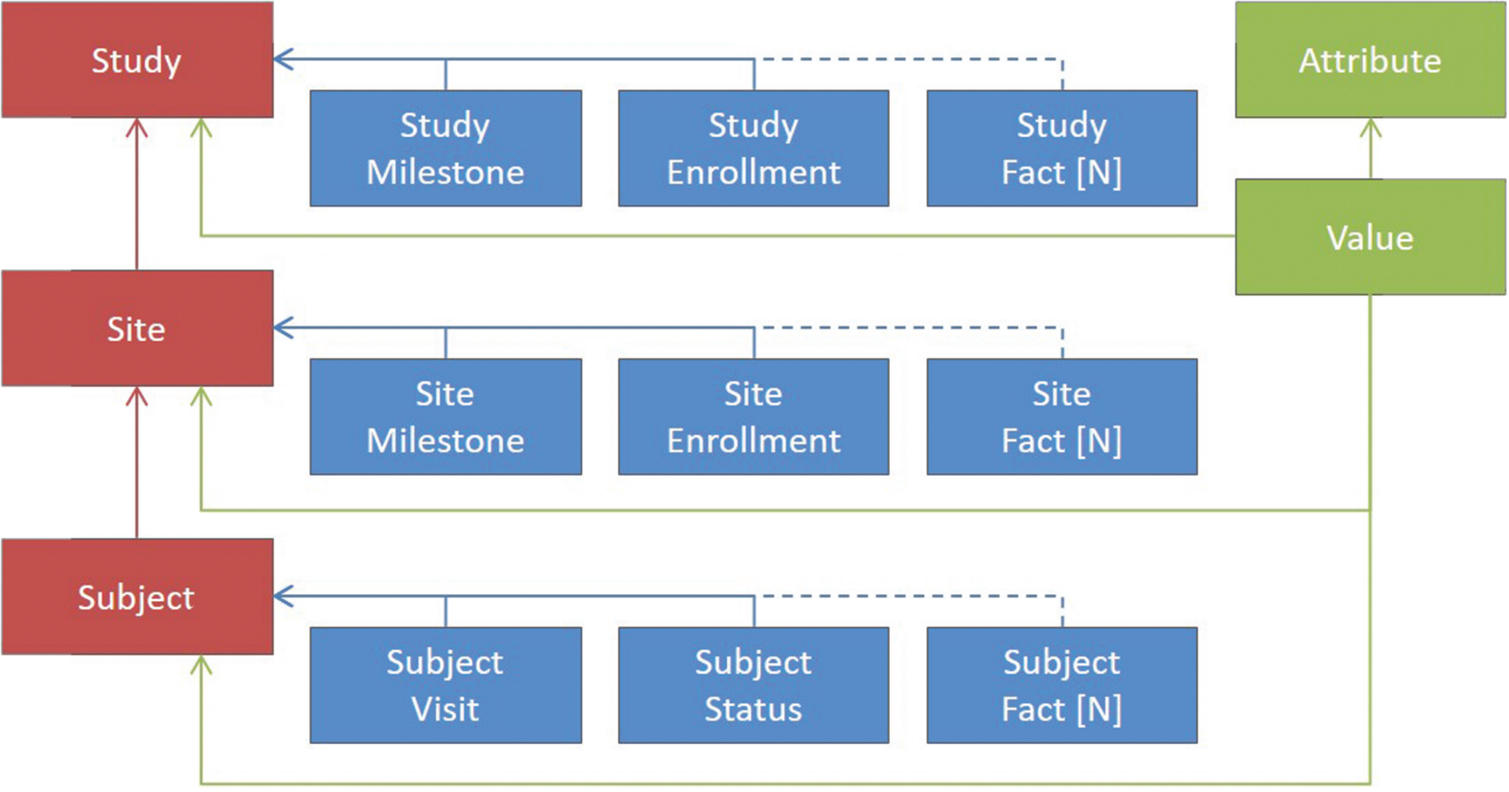
Simplified representation of the operational data model.

Figure 2 shows a greatly simplified schematic representation of the ODW design principles, as the actual number of tables is greater than what could be legibly represented in a figure. In red are examples of some key dimensions in the warehouse, such as clinical studies, sites and subjects. Between the dimensions are relationships that need to be maintained for data integrity. For each of the dimensions, there are a number of facts associated with them, depicted in blue. These facts depend on the entity in question, although some facts, such as milestones, serve a similar purpose across multiple dimensions. For these dimensions and facts, a canonical set of columns has been defined that represents the important entities that we have seen in common business practice. This traditional approach to data warehousing offers a number of advantages. It allows database constraints to prevent a variety of data quality issues, provides efficient storage for high volumes of data and offers easily interpretable records for communications and problem solving.

In addition to the model features described above, where all of the features of the facts are known, we have introduced the ability to load data in an entity-attribute-value (EAV) representation, shown by the items in green. This offers the flexibility to ingest unexpected or rarely occurring entities that are needed for trial operations without the overhead of making changes to our database schema or extraction, transformation and loading (ETL) components. On the loading side, we have introduced a configuration UI, tables that hold configured values for file types and expected columns and a file loader that transforms tabular input into the unpivoted storage format. Each record loaded in this way is associated with one of our core dimensions to maintain data quality. Examples of the types of entities included in the model are Account, Product, Study, Region, Country, Site, Subject, Contact, Address, Milestone, Visit, Report, Payment, Issue, Page, Query, Deviation, Regulatory, Document, Forecast and others.

### ETL and master data

Before the data are brought into the landing area for processing, they are checked for referential integrity (data for unknown studies are rejected, records for unknown sites are flagged, etc.). This is necessary so that data from multiple sources can be successfully linked and the integrity and security of the data can be enforced. Therefore, master data on studies, sites, accounts, etc. is an essential data feed for ODW. The best source for this data is an enterprise master data management (MDM) system that drives standardization of key entities throughout the company. Internally, we have developed this capability for a variety of common entities, such as trials, investigators and institutions. If a formal MDM feed is not available, the CTMS can be used as a surrogate.

In the staging database, these sources are merged into a common vocabulary of key entities, and the incremental feeds are decoded into the latest snapshot. The ODW is a dimensional SQL Server database that keeps both the current records as well as a history of updates for all information. While the ODW captures information from EDC systems important to operations, such as patient status and adverse event counts, a full mapping of trial complexity is left for the CDW (19).

The ODW is refreshed at prescribed intervals through fully automated ETL feeds from the various source systems, nearly always on a frequency of 24 hours or less. The ETL has been implemented in Informatica PowerCenter (20) and Business Objects Data Stage (21). All sources first reach the landing area of our database in formats closely resembling the incoming format, are transformed and conformed in the staging area and moved to the warehouse for reporting and analytics. The database stores the full history of updates, allowing retrospective and diagnostic analysis of historical records. Consuming applications may either access data directly through versioned views that shield applications from minor modifications in the data model or using derived metrics as described below.

For the ODW to be comprehensive, it must capture results from the common transactional systems used for administering and collecting data from clinical trials. The CTMS is typically the repository where many of the key entities are originally captured, including the studies, countries and sites used in the trials, the investigators and the clinical institutions where they see patients, the clinical monitors who train and oversee site activities, a variety of reports capturing assessments of site activity and monitor visits and milestones at multiple levels. While there are a large number of common industry practices that result in similar data structures, the business rules employed at individual organizations are often reflected by subtle changes in the data storage. For example, the exact fields used to describe a protocol deviation and its follow-up may differ, as well as whether a protocol deviation is stored distinctly as its own entity or a subtype of a more general clinical issue. Typically, CTMS systems are built with a relational database as an underlying data store. As such, integration of the data from a CTMS is the result of the extraction of incremental records from the system to a common interchange format, followed by conformation of data at the entire system level, as trial-to-trial variations are usually small or fall within a few prescribed areas. Some examples of these would be varying templates for the questions recorded during site-monitoring visits or custom milestones recorded for sites or studies.

As stated above, two other crucially important data sources are the EDC system used to record the bulk of physician-collected information at the investigational sites and the IRT system that tracks information necessary for patient randomization and drug dispensation. The data structures for both of these systems vary per clinical protocol due to the visit schedule and data elements that are collected at each visit. Using the web service connections provided by the leading EDC vendors provides a way to retrieve all transactions from the systems incrementally in CDISC ODM format (22). That feed includes both clinical and operational data elements, such as queries against data items, source data verification status, review status and page locking and freezing. Having all of the data together in our warehouse allows for the creation of rules that define the precedence of each system’s data in computing derived entities. For example, data in EDC, which is entered and reviewed by physicians and queried by both site monitors and data review teams, is typically viewed as being of the highest quality, but its entry may be delayed compared to other sources. This also allows the complete picture of a patient’s activity to be assembled in cases where particular visits may not have a collection point in all systems.

### Metrics and indicators

Monitoring clinical trial performance involves periodic review of operational metrics and KPIs, KQIs and KRIs. Operational metrics are typically aggregated quantities derived from routine operational data and serve to identify and mitigate risks, monitor and improve operational workflows, manage resource allocation, strengthen investigator and sponsor relationships and other purposes. There is substantial variability in metric requirements depending on individual study needs, process differences, sponsor preferences and source system capabilities. Thus, a comprehensive solution for operational reporting must be able to support a variety of metrics, KPIs, KQIs and KRIs and allow flexible and expedient definition of additional metrics, as needed.

**Figure 3:**
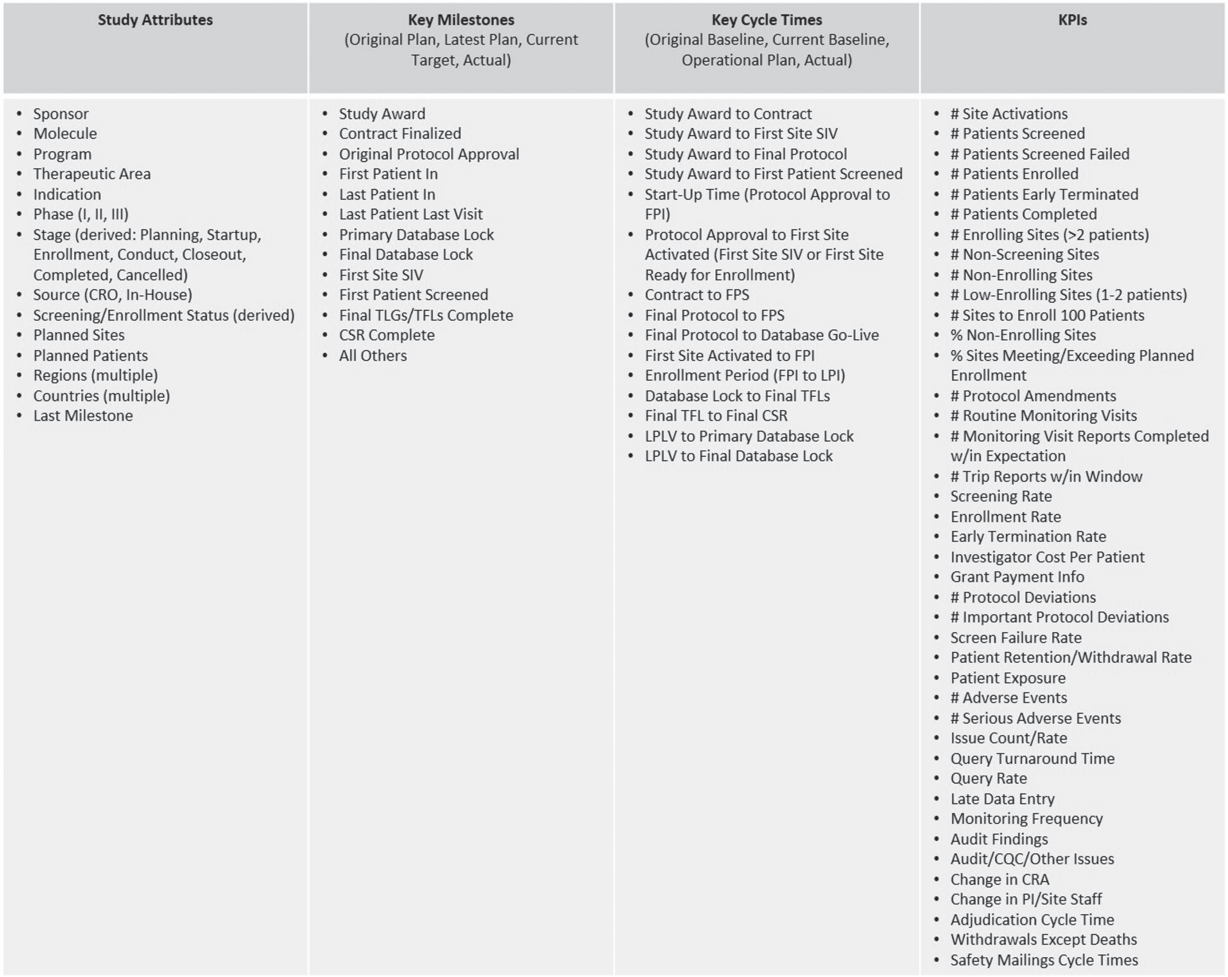
Representative study and portfolio metrics and attributes.

The majority of the operational metrics and KPIs available in ODW are common across studies and programs and are driven by the need for standardized study reporting and portfolio governance. Typical operational metrics reflecting site activation, subject enrollment, issue management, data collection, etc. are an integral part of our operational data model described above. Figure 3 shows a listing of representative metrics that are computed at the level of clinical studies. These consist of key attributes, which descriptive of the nature and type of the study; key milestones that show the progress of the trial from inception through execution to completion; cycle times, which measure the rate of progress between milestones; and KPIs that measure the amount of activity and quality of work in the study. The framework allows for roll-ups and average values at multiple levels of aggregation, per subject, site, study, country, etc., to be pre-calculated as part of the standard ETL process. These are available for standard and *ad hoc* reporting through direct database access and APIs.

To support the continuously evolving operational reporting and RBM needs, we have developed a generalizable approach for calculating additional arbitrary metrics and risk indicators as part of the ETL schedule. Our system enables users to define aggregate metrics and derived quantities using arbitrarily complex SQL expressions, derive them from any data source that is brought into ODW, parameterize them, group them in reusable templates and expose them to the UI and data visualization layer. Further, our system allows us to easily configure the source-to-target mapping of the data fields that ultimately turn into metrics, making it highly robust, generalizable and extensible.

**Figure 4:**
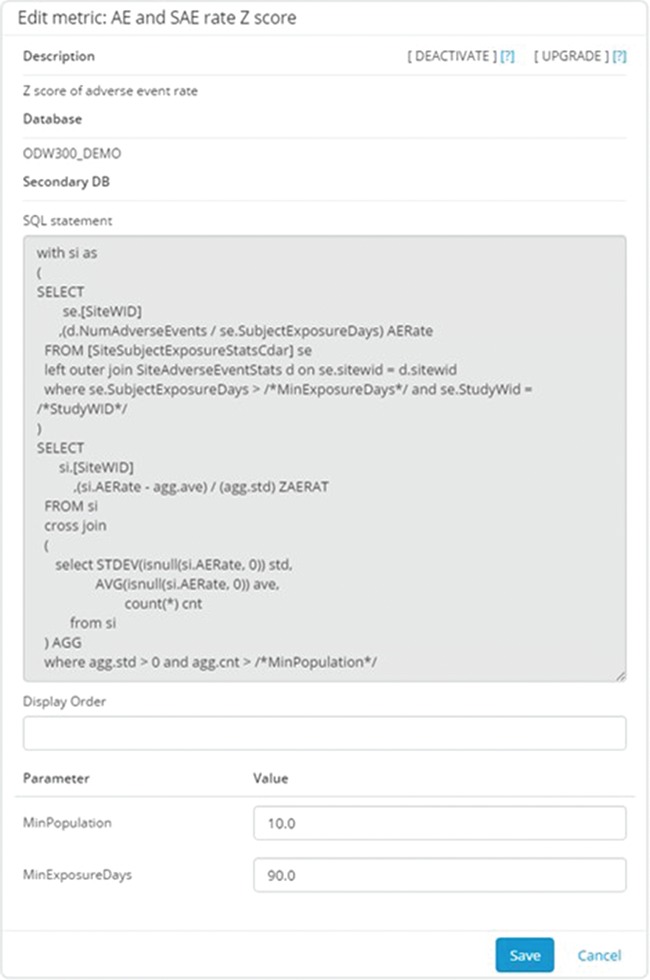
Metric configuration UI. This application allows the configurators to specify the parameters stored in the system to describe and implement the metric. Metrics can be defined using arbitrarily complex and parameterized SQL statements. The parameters are entered separately to promote reuse of the overall logic.

Figure 4 shows an example of the configuration interface for an individual metric. Each metric has a short name for use in user displays and a longer description for greater clarity on the meaning and its derivation. The technical definition of the metric consists of three parts: a server definition, an SQL statement and a list of parameters. The server definition offers the flexibility to retrieve data from the main operational warehouse or from other application-specific marts that may provide additional data. The SQL statement allows for complex logic to be encoded along with selection of the relevant data fields, provided that the return structure includes the identifier of the site of interest in the first returned column and the data item being reported in the second one. Parameters allow for commonly variable elements of the definition logic to be demarcated and substituted in the SQL statement, allowing for greater maintainability. Typical examples of such parameters include numeric limits for data ranges, such as the number of days used to define recent changes in site staff. Our Portfolio Reporting application, described in detail later, takes extensive advantage of this framework to allow additional metrics to be defined without requiring a change to the database schema or application code.

**Figure 5:**
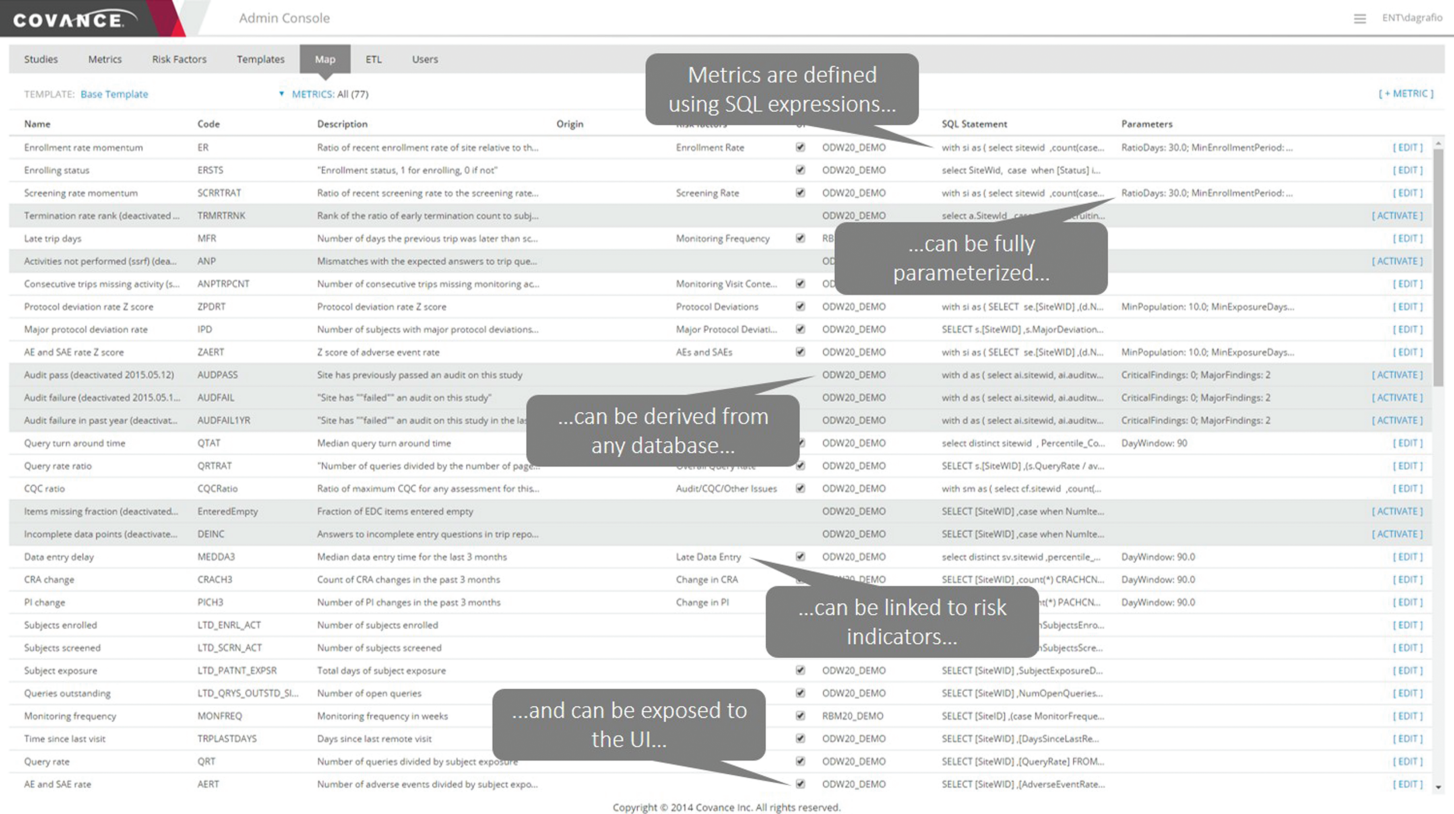
Xcellerate Monitoring Administration Console. The interface allows a set of metrics to be edited and configured into templates for use across sets of studies.

**Figure 6:**
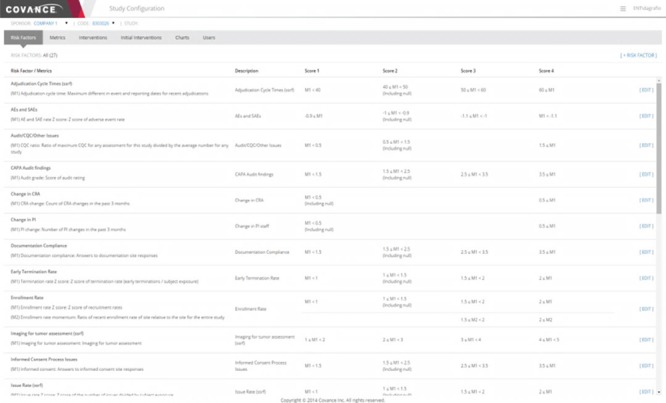
Representative screenshot of the Xcellerate Monitoring Study Configuration Console. The study configuration console allows study-specific updates to be made for individual metrics, including changing parameterized values and thresholds for scoring the risk levels. The particular example illustrated below shows the rules used to define various risk indicators used in RBM.

Metrics and risk indicators are managed through the Xcellerate Monitoring Administration Console (Figure 5) and Study Configuration Console (Figure 6). These tools are used to define metrics and risk indicators that are relevant to the trial at hand, set the appropriate thresholds, triggers and aggregation functions that convert metrics into risk levels and monitoring interventions and assign authorized users and roles.

The Administration Console (Figure 5) provides functionality to create and maintain a central library of indicators and promote consistency across trials. This is done by assembling settings into templates that can be propagated to individual studies with a single click on the UI. A key part of the template is the list of metrics that are chosen to be calculated, which may contribute directly to the logic for risk indicators or be brought in to provide additional information about site performance. The templates include default logic and thresholds for risk indicator calculations. The Administration Console also has functionality for assigning roles and user permissions in individual studies.

While there are considerable advantages to keeping a consistent library of indicators, such as minimizing training for study teams and allowing cross-trial comparisons, it is also important that users have the ability to adapt the template values for particular trials. The need for customizations can be reduced by using appropriate normalizations for value thresholds and/or the distribution of site values in the trial as a comparison but cannot be completely eliminated. These per-study adjustments are enabled by the Study Configuration Console (Figure 6) and can be in several different areas. For unique data sources or risk concerns, study-specific metrics and risk indicators can be defined. For trials where RBM is employed, a monitoring plan would define the specific activities that would be required at each intervention level, such as the frequency of onsite visits and the percentage of source data verification required. The Study Configuration Console offers a single point of input of these values for the entire integrated system, thus eliminating duplicative work and simplifying the communication of the resulting indicators and signals to site monitors and other authorized members of the study team. This interface also provides for the selection of default values for display in visualizations as well as user administration within a particular trial.

### APIs

The ODW is accessed by a suite of visually rich and intuitive UIs through RESTful web services APIs. These APIs also make it possible to access the data through programmatic means or through alternative data visualization and analysis tools, such as Spotfire (23), Tableau (24), R (25), SAS (26), etc. We have developed a set of APIs that support a variety of client exports. In order to meet the specifications for our partners, we have introduced the ability to derive fields and milestones and format XML definitions through a configuration interface. This allows us flexibility to implement data quality specifications and support multiple formats for the same data without creating multiple hard-coded endpoints.

## Results and discussion

Operational data is used by many different user groups to support a wide range of decisions around project management, portfolio governance and management oversight. In the Xcellerate suite, access to the metrics and KPI/KQI/KRIs stored in the ODW is provided mainly through the Xcellerate Study Reporting, Portfolio Reporting, Risk Review and clinical research associate (CRA) dashboards.

### Study and portfolio reporting

**Figure 7:**
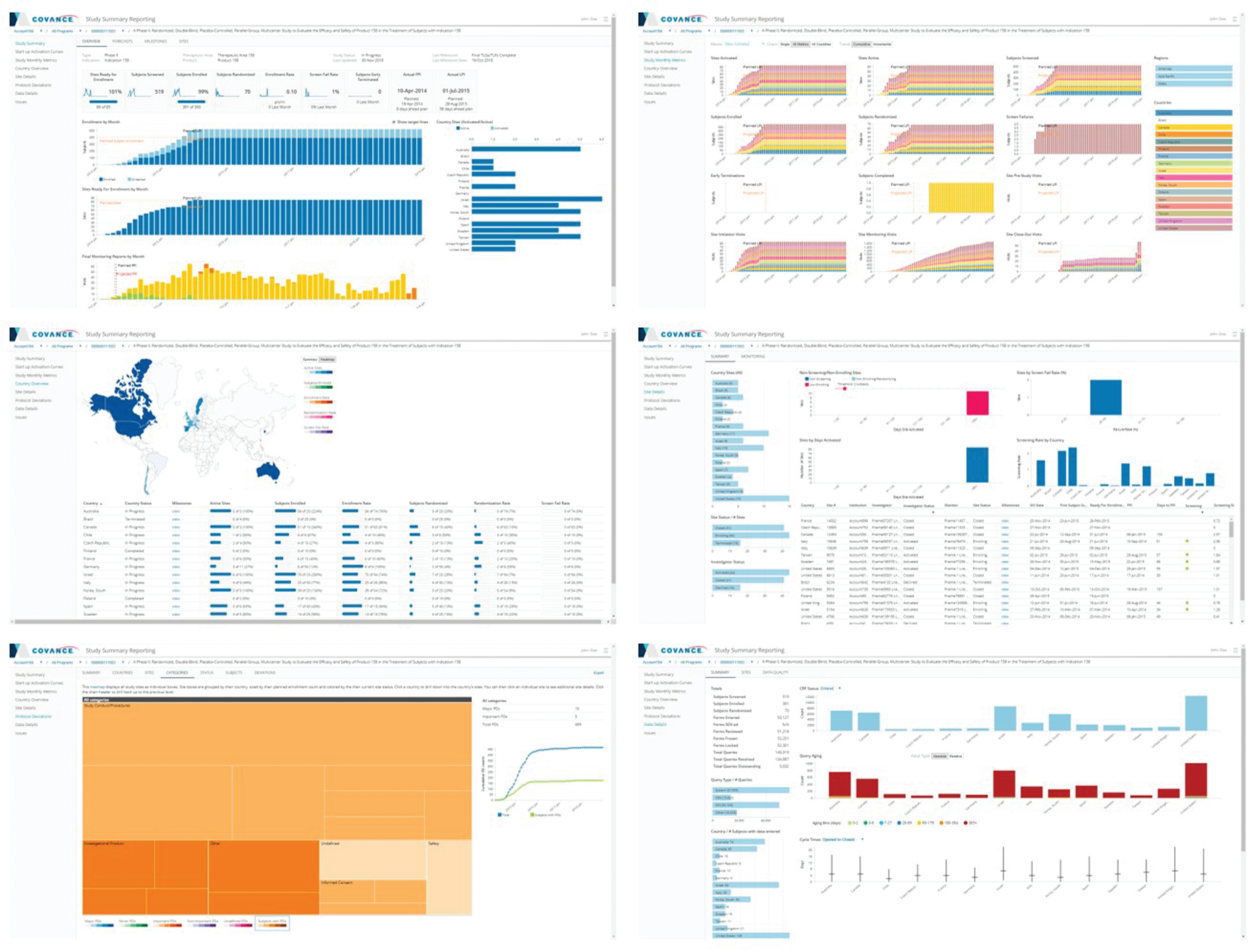
Representative screenshots of the Study Reporting UI that uses the ODW as a source. Top left: study summary view. Top right: monthly metrics view. Middle left: country view. Middle right: site view. Bottom left: protocol deviations view. Bottom right: data management view.

The Xcellerate Study Reporting dashboard (Figure 7) is designed to enable project teams to track the progress of their trials against milestones and performance targets. This interactive dashboard offers longitudinal views of KPIs and metrics at the individual study level, with extensive drill-down, filtering and sorting capabilities. The dashboard is organized into several views, each reporting key metrics related to the performance of clinical development functions, sites, geographical regions or combinations thereof. The study summary view provides visibility into whether key performance objectives such as site activation, subject enrollment and study completion dates are on track. It also includes key milestones and projections for meeting study objectives given the observed trends. The site startup and subject enrollment view provides up-to-date information along with monthly trends on the number of sites activated, the number of site activation, initiation and close-out visits, the number of subjects screened, enrolled, terminated and completed and many others. The view provides filtering capabilities by country and region and allows for cumulative or incremental displays. The dashboard also provides so-called S-curve charts that show the cumulative number of sites that have reached a particular milestone compared against the initial and current plan (e.g. Institutional Review Board approval, contract execution, green light approval, site initiation visit, etc.). The site performance view provides information on site status, site subject enrollment and key site milestones. Sites can be filtered and compared by geographic location, investigator, monitor, activation date or any other relevant attribute. Several temporal trend views are also provided to help the user focus on recent developments. For assessing country performance, we compare country milestones, site activation, subject enrollment and screen failure rates versus target. A world map allows geo-mapping of any desirable metric to enable quick detection of regional patterns. The view can also be sorted by any metric to identify leaders and laggers. The protocol deviation view provides insights into protocol deviation accrual for study management and regulatory reporting purposes. All protocol deviations are classified by importance, category and resolution status. Each protocol deviation can be traced to the site and subject with extensive drill-down to any level of resolution, and details around protocol deviations can be exported as a comma separated values (CSV) file for further processing. Additional views offer insights into data management metrics, such as number of pages submitted, percent of source document verification, number of outstanding queries, query ageing, data entry timelines, etc. As with other views, the information can be dissected by country, query time and site. Bubble charts provide visualizations of data collection and data quality trends by country and region.

**Figure 8:**
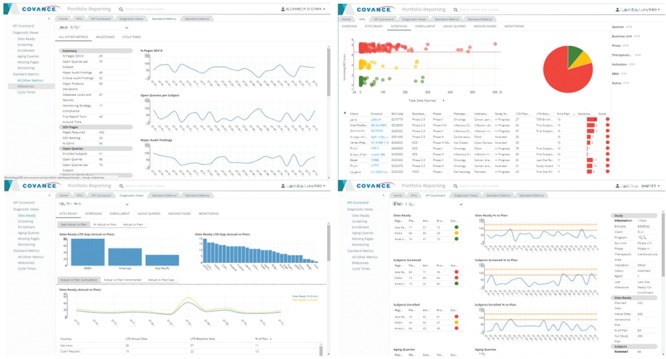
Representative screenshots of the Portfolio Reporting UI created with metrics derived from the ODW. Top left: milestones. Top right: KPIs. Bottom left: site readiness. Bottom right: KPI scorecard.

Recognizing that project management and portfolio governance have very different reporting needs than monitoring the progress of an individual trial, we have developed a highly configurable, web-based portfolio dashboard (Figure 8) designed to provide a comprehensive view of the health of a portfolio of studies at any desirable level of aggregation (therapeutic area, clinical indication, phase or any arbitrary grouping of studies selected by the user, as long as they have the appropriate access rights). The portfolio dashboard uses the same generalized, extensible framework for KPI, milestone and cycle time reporting, allowing users to view the status on key deliverables, past performance on completed activities, current position and risk of activities not yet completed, proposed key deliverables, geographic footprint overlaid with site performance and other start-up, data management, financial and quality metrics. It is based on a generic attribute model and features a UI that supports configurable interactive reports composed of multiple figures and tables, consistent attribute filtering and drill-down across all views and *ad hoc* analytics based on a customizable charting library.

### Central and site monitoring

One of the main uses of the ODW is to support RBM (16). For RBM risk review, data are transferred from the ODW for each individual study based on the schedule established by the RBM team. Certain core information, such as sites, monitors and physicians, are brought for every trial in a standard structure. Configuration allows per-trial choices for the types and implementation for the metrics, calculated for each site at each time point, with scoring rules allowed to be customized for the needs of the monitoring team. This allows new risk indicators to be incorporated as data become available and deemed appropriate for use. The computed risk scores are translated into recommendations for changes in site monitoring, which are reviewed by the RBM lead (16).

At the core of the overall approach is the creation and technical implementation of individual risk indicators. This begins with a crisp definition for the exact metric or set of metrics, which in our experience is best done in a collaborative environment that takes input from the RBM leads, site monitors and other members of the study team, leveraging historical data from the warehouse for similar studies. The next step is the identification of the source data, and one of the operational warehouse’s primary goals is to make this as easy and consistent across trials as possible. The range of values that drive thresholds for converting metrics into risk levels generally falls into two categories: static limits that are typically used to drive behavior of site-monitoring staff such as the time to data entry or relative values for sites within the study that are expected to be strongly dependent on the particular trial such as the rate of adverse event reporting. Technically, the KRI calculation can be coordinated with the ETL automation that drives the update of data in the warehouse or triggered by user input. All values for metrics and risk indicators are stored in the database.

The Risk Review application is principally aimed at central monitors. A separate interface, the CRA Dashboard, provides CRAs with mobile and web access to all the information they need to perform their site-monitoring activities in an efficient and effective manner. The CRA Dashboard provides an elegant interface that offers real-time access to site data and enhanced visibility of site and country performance to improve monitoring visit strategy and compliance, reduce email volume, enhance productivity and enable proactive risk management and timely intervention and ultimately improve CRA behavior, site management and patient care.

**Figure 9:**
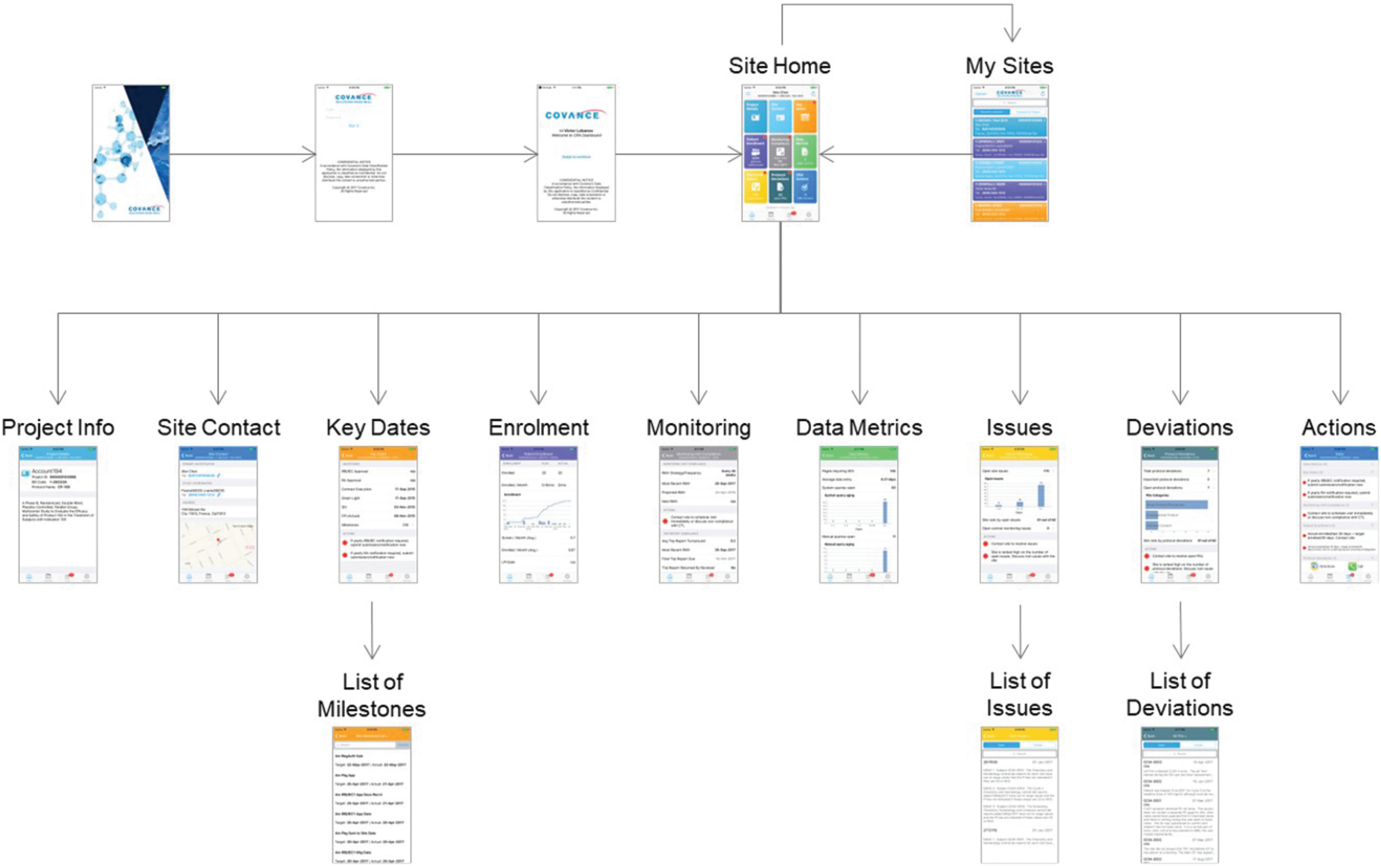
CRA Dashboard (mobile version).

The overall design of the screen navigation for the mobile CRA dashboard is illustrated in Figure 9. After authentication, the site monitor is presented with a listing of the trials and clinical sites that they are responsible for, including an indication of the number of alerts for each one. Upon selecting a particular trial and site, the user is directed to the Site Home screen that lists all the possible details that can be selected, including badges for alerts for each item. The Project Info screen shows high-level information about the protocol, including the sponsor and protocol synopsis. The Site Contact tab shows the primary investigator and study coordinator’s information, including the phone number, address and a map locating the site. The Key Date screen shows the primary milestones related to feasibility and site startup. The Enrollment tab shows the current screened and enrolled patients, including trending. The Monitoring tab shows the scheduled frequency of visits, when the last and next visits should occur and compliance for filing trip reports. The Data Metrics tab shows outstanding work to be done in the EDC system, such as the number of pages remaining for source data verification, the number of outstanding queries and the length of time that queries have been open. The open issues and required actions are shown on the Site and central monitoring (CM) Issues tab with drill-down available into the details of each item. Similarly, the Deviations tab lists protocol deviations and provides full information for each, with the ability to filter by categorization and importance. Finally, the Actions/To Do tab gives a summary of all of the outstanding items from all of the other screens.

### *Ad hoc* reporting

As stated above, the Xcellerate platform comes with a comprehensive set of RESTful web services APIs that allow programmatic access to all the data and metadata stored in the ODW. Additional reporting needs that are beyond the scope of the standard reporting UIs can be implemented using standard business intelligence tools. Most of these tools are able to consume RESTful APIs, retrieve the data of interest, perform additional aggregation and filtering and deliver it to the end users in a desirable visual representation. Exploratory data mining and *ad hoc* analysis of the operational data is supported through direct access to the ODW schema. Popular analytics tools such as R and SAS can query the underlying SQL Server database directly using custom SQL queries. More sophisticated *ad hoc* reporting/analytic needs can be further facilitated through the development of custom data marts.

### SQL versus NoSQL

It is important to note that there are several market offerings that claim to solve operational reporting needs through the use of Hadoop, NoSQL, data lakes and other ‘big data’ approaches (27–29). We believe that the technologies themselves are sound, but their use for operational reporting is premature and misguided. The advantage of non-relational approaches, such as data lakes, is that they can ingest large amounts of data very efficiently while postponing data mapping/normalization until query time. While this is true in principle, the effort of data mapping/normalization still has to occur. Given that the majority of operational reports are standardized, it is more efficient to normalize the data upfront and thus simplify the effort at reporting time. Moreover, query and reporting capabilities for these technologies are still evolving and are not as powerful as the use of SQL in relational data stores (19).

## Conclusion

The ODW presented herein combines the maturity and robustness of a relational database, the performance of a denormalized dimensional data model, the flexibility of a configurable metrics engine and the convenience of a web-based UI to deliver timely insights into the operational health of clinical trials. Coupled with the CDW, the ODW and associated processes minimize the delay in mapping data coming from investigational sites; enables timely review and intervention by monitoring staff; reduces the workload for data management, biostatistics, programming and clinical teams; and brings important practical benefits across a wide range of clinical and translational applications.

## Contributors

M.A.F. conceptualized the invention, led the design, development and implementation of the software and drafted the initial manuscript. L.M., M.A., P.K., J.C. and V.S.L. contributed to the design, development and implementation of the software and contributed to the manuscript. D.K.A. was accountable for the work, contributed to the manuscript and approved the final manuscript as submitted.
